# The pattern of retinal ganglion cell dysfunction in Leber hereditary optic neuropathy

**DOI:** 10.1016/j.mito.2017.07.006

**Published:** 2017-09

**Authors:** A. Majander, A.G. Robson, C. João, G.E. Holder, P.F. Chinnery, A.T. Moore, M. Votruba, A. Stockman, P. Yu-Wai-Man

**Affiliations:** a UCL Institute of Ophthalmology, London, UK; b Moorfields Eye Hospital, London, UK; c Department of Ophthalmology, University of Helsinki, Helsinki University Hospital, Helsinki, Finland; d MRC-Mitochondrial Biology Unit, Cambridge Biomedical Campus, Cambridge, UK; e Department of Clinical Neurosciences, Cambridge Biomedical Campus, University of Cambridge, Cambridge, UK; f Ophthalmology Department, UCSF School of Medicine, San Francisco, CA, United States; g School of Optometry and Vision Sciences, Cardiff University, and Cardiff Eye Unit, University Hospital Wales, Cardiff, UK; h Wellcome Trust Centre for Mitochondrial Research, Newcastle University, and Newcastle Eye Centre, Royal Victoria Infirmary, Newcastle upon Tyne, UK

**Keywords:** Leber hereditary optic neuropathy (LHON), The pattern electroretinogram (PERG), The photopic negative responses (PhNR), Critical flicker fusion, Spatial contrast sensitivity, Chromatic resolution

## Abstract

Leber inherited optic neuropathy (LHON) is characterized by subacute bilateral loss of central vision due to dysfunction and loss of retinal ganglion cells (RGCs). Comprehensive visual electrophysiological investigations (including pattern reversal visual evoked potentials, pattern electroretinography and the photopic negative response) performed on 13 patients with acute and chronic LHON indicate early impairment of RGC cell body function and severe axonal dysfunction. Temporal, spatial and chromatic psychophysical tests performed on 7 patients with acute LHON and 4 patients with chronic LHON suggest severe involvement or loss of the midget, parasol and bistratified RGCs associated with all three principal visual pathways.

## 1 Introduction

Leber hereditary optic neuropathy (LHON) (OMIM 535000) is a primary mitochondrial DNA (mtDNA) disorder that presents with bilateral subacute loss of central vision (Nikoskelainen et al., 1996, Yu-Wai-Man and Chinnery, 2013, Yu-Wai-Man et al., 2014). The majority of patients harbour one of three common mtDNA mutations (m.3460G > A in *MTND1*, m.11778G > A in *MTND4* and m.14484T > C in *MTND6*) that affect complex I subunits of the mitochondrial respiratory chain (Mackey et al., 1996). Despite the universal cellular role of mitochondria, retinal ganglion cells (RGCs) within the papillomacular bundle are particularly severely affected accounting for the characteristic dense central or caecocentral scotoma in this disorder. Although the underlying pathological process is still not fully defined, this tissue specificity has been ascribed to an increased vulnerability of RGCs to both disturbed mitochondrial energy metabolism and the increased formation of reactive oxygen species (Carelli et al., 2004a, Carelli et al., 2004b, Lin et al., 2012, Sadun et al., 2000, Levin, 2015, Sadun et al., 2015). LHON shows maternal inheritance, but there is variable disease penetrance and a marked sex bias with about 50% of male carriers losing vision during their lifetime compared with about 10% of female carriers (Mackey et al., 1996).

The histopathological observation of loss of the small calibre axons that constitute the papillomacular bundle was originally observed on histopathological sections of post mortem optic nerve samples obtained several decades after disease onset (Sadun et al., 1994, Kerrison et al., 1995, Sadun et al., 2000). More recently, in vivo studies involving high-resolution optical coherence tomography (OCT) have revealed a major loss of the temporal peripapillary nerve fiber (RNFL) and macular RGC layers within 3 months of disease onset. Interestingly, pathological thinning within the macular RGC layer was an early sign that was already apparent in the presymptomatic phase (Barboni et al., 2005, Barboni et al., 2010, Akiyama et al., 2013, Zhang et al., 2014, Mizoguchi et al., 2015, Balducci et al., 2015). Following disease conversion, swelling of the peripapillary RNFL spreads circumferentially from the inferotemporal segment of the optic disc to involve the remaining quadrants, before RNFL atrophy becomes established within 3–9 months after the onset of visual loss. Hyperemia and fluctuating mild swelling of the prepapillary RNFL can also be seen in unaffected LHON carriers without visual loss or progression to full disease conversion (Nikoskelainen et al., 1982).

The function of the papillomacular bundle may be assessed objectively using pattern reversal visual evoked potentials (PR-VEP) and pattern electroretinography (PERG). Reported PR-VEP abnormalities in LHON are consistently severe, but the utility of the PERG in LHON is more controversial and there are conflicting reports in the literature regarding the timing of responses and the sequence of losses (Nikoskelainen et al., 1977, Hrynchak and Spafford, 1994, Mashima et al., 1997, Sharkawi et al., 2012, Ziccardi et al., 2013, Ziccardi et al., 2015, Jarc-Vidmar et al., 2015). The full-field photopic negative response (PhNR) has been used to assess generalized RGC function in glaucoma and other acquired optic neuropathies (Machida, 2012, Morny et al., 2015), but there are no published studies of PhNR in LHON. The applicability of PhNR as a potential objective functional index of RGC function in LHON therefore warrants further investigation.

RGCs are classified into the three major subtypes of RGCs, namely midget, parasol and small bistratified ganglion cells, which are thought to contribute to the parvocellular, magnocellular and koniocellular pathways, respectively. These distinct RGC populations and their associated pathways can be tested by modifying standard psychophysical measures. In general, the processing of high spatial frequency information has been linked with the parvocellular pathway whereas high temporal frequency information is thought to be integrated by the magnocellular pathway. Red-green processing and blue-yellow processing have been linked with the parvocellular and koniocellular pathways, respectively. Parallel processing in the retina and visual pathways have been the subject of several recent comprehensive reviews (e.g., Dowling, 1987, Wässle and Boycott, 1991, Rodieck, 1998, Lennie and Movshon, 2005, Lee et al., 2010, Dacey et al., 2014).

LHON is thought to mainly affect midget RGCs, which have the smallest calibre axons and are the predominant subtype within the papillomacular bundle, mediating visual information including high spatial frequencies and red-green chromaticity (Sadun et al., 1994, Hrynchak and Spafford, 1994, Kerrison et al., 1995, Sadun et al., 2000). In contrast, another melanopsin-expressing RGC subtype appears relatively preserved and this peculiarity likely accounts for the frequently preserved pupillary light reflexes, even in severely affected LHON patients (Kawasaki et al., 2010, La Morgia et al., 2010). One previous report indicated mild impairment of the magnocellular pathway in unaffected LHON carriers (Gualtieri et al., 2008), but there are no robust data regarding the involvement of the parasol and small bistratified RCG subtypes in LHON.

In this study, we investigated the pattern of RGC dysfunction in a well-phenotyped cohort of LHON patients in both the acute and chronic phases of the disease by using a comprehensive visual electrophysiological and psychophysical assessment protocol. Our aim was, firstly, to characterise the electrophysiological responses to better define the phenotypic features of LHON and to establish the most appropriate methods for monitoring RGC function and disease progression objectively. Secondly, we used psychophysical tests of temporal, spatial and chromatic vision to investigate the relative involvement of distinct RGC populations in the pathophysiology of LHON.

## 2 Methods

### 2.1 Subjects

This was a prospective case study of 12 affected patients (A1–A12) and 9 unaffected carriers (U1–U9) harboring one of the three common mtDNA LHON mutations (Table 1). In addition, retrospective visual electrophysiological data for 5 affected LHON patients (A13-A17) were retrieved from the hospital database of Moorfields Eye Hospital, London, UK. In total, there were 4 affected female and 13 affected male patients. Affected LHON patients and unaffected LHON carriers in our cohort underwent an ophthalmological examination that included the following investigations as indicated in Table 1: best corrected visual acuity (BCVA) assessment using the Early Treatment Diabetic Retinopathy Study (ETDRS) chart; slit lamp examination; automated Humphrey visual field perimetry (Program 30-2, Humphrey Visual Field Analyzer, Model 750, Humphrey Instruments, San Leonardo, CA); and optical coherence tomography (OCT) imaging of the macula and the optic nerve head (see details below).

**Table 1:** Clinical characteristics of the recruited LHON cohort and the timing of investigations in relation to disease onset.

Subjects	Age (Y) sex	Genotype	BCVA logMAR^a^		Visual field defect MD (dB)		Time from LHON onset at study (months)				
							OCT		Electrophysiology		Psychophysics
			RE	LE	RE	LE	RE	LE	RE	LE	RE/LE
	Affected LHON patients										
A1	42 M	m.11778G > A	1.4	1.5^a^	− 32.26	− 28.63^a^	15	15	16	16	9 LE
A2	18 M	m.11778G > A	1.6	1.5^a^	− 32.07	− 32.89^a^	NP		18	18	10 LE
A3	19 F	m.3460G > A	1.9^a^	1.9	− 34.39^a^	− 34.46	0.7	0	17	16	10 RE
A4	21 M	m.11778G > A	2.0	2.0^a^	− 34.18	− 33.79^a^	15	19	15	19	9 LE
A5	17 M	m.11778G > A	2.2^a^	2.28	− 34.48^a^	− 34.31	10	10	14	15	10 RE
A6	15 M	m.11778G > A	1.6^a^	1.7	− 29.11^a^	− 28.56	6	6	8	8	6 RE
A7	22 M	m.11778G > A	0.7	0.8^a^	− 19.36	− 15.10^a^	32	32	NP		36 LE
A8	33 M	m.11778G > A	2.1^a^	2.1	− 33.30^a^	− 33.23	34	28			34 RE
A9	24 M	m.14484T > C	1.3^a^	1.3	− 12.01^a^	− 12.63	20	23			23 RE
A10	51 F	m.11778G > A	1.6^a^	1.6	− 24.23^a^	− 27.51	40	40			40 RE
A11	23 M	m.11778G > A	1.8^a^	2.3	− 32.11^a^	− 29.76	4	5			4 RE
A12	50 F	m.11778G > A	1.9	1.9	− 30.54	− 29.86	6	0	10	3	NP
A13	22 M	m.11778G > A	0.8	1.0	NP		0	1	0	1	
A14	36 M	m.14484T > C	1.3	1.0			98	98	5	5	
A15	7 M	m.11778G > A	0.6	2.0			140	140	27	27	
A16	7 F	m.3460G > A	1.7	1.5			NP		2	2	
A17	59 M	m.3700G > A	1.9	1.9					20	20	

	Unaffected LHON carriers										
U1	44 F	m.11778G > A	0^a^	0	− 10.83^a^	− 18.08	P		NP		P
U2	46 M	m.11778G > A	0^a^	0	− 2.57^a^	− 2.42					
U3	52 F	m.3460G > A	0.1^a^	0	− 0.97^a^	− 1.43					
U4	47 F	m.11778G > A	− 0.1^a^	− 0.1	− 3.51^a^	− 3.17					
U5	50 F	m.14484T > C	0.1^a^	0	0.18^a^	0.26					
U6	50 F	m.11778G > A	− 0.1^a^	0.1	− 2.59^a^	− 0.24					
U7	41 F	m.3460G > A	0^a^	0	0.06^a^	− 0.99					
U8	60 F	m.3460G > A	− 0.1^a^	0	1.11^a^	0.22					
U9	46 F	m.11778G > A	0^a^	0	− 1.81^a^	− 1.64					

Abbreviations: BCVA, best corrected visual acuity: DOA, dominant optic atrophy; F, female; LE, left eye; LHON, Leber hereditary optic atrophy; M, male; NP, not performed; P, performed; RE, right eye.

a BCVA and MD of the eye used for the monocular psychophysics tests are indicated by asterisks.

The normal subjects for psychophysical tests were 15 individuals aged 17 to 78 years old at the time of testing with normal BCVA and normal color vision as assessed by standard color vision tests. Only 12 of the normal subjects had their L-cone temporal contrast sensitivities measured. Written informed consent was obtained from all subjects or their guardians. The study was approved by the local ethics committees at Moorfields Eye Hospital and University College London and it conformed to the standards of the Declaration of Helsinki.

### 2.2 Optical coherence tomography (OCT) imaging

The Spectralis™ platform (Heidelberg Engineering Ltd., Heidelberg, Germany) was used for SD-OCT imaging of the macula and the optic nerve head. Automated segmentation and thickness analyses were performed for perifoveal volumetric retinal B-scans using the Heidelberg Engineering segmentation tool, included in the Spectralis Glaucoma Module software (version 6.0). Of the 10 retinal layers that were automatically defined and manually confirmed, the following thickness values were recorded from the four sectors of the inner ring (between 1 and 3 mm diameter) of the nine macular ETDRS subfields as described elsewhere (Majander et al., 2016): (i) retina, (ii) retinal nerve fiber layer (RNFL), (iii) combined GCL and inner plexiform layer (IPL), (iv) inner nuclear layer (INL), (v) outer plexiform layer (OPL), (vi) combined OPL and outer nuclear layer (ONL), and (v) inner retina. The thickness of the outer retinal layers was calculated by subtracting the thickness of the inner retinal layers from the total retinal thickness. Normative data was generated from SD-OCT images of 48 healthy eyes of 48 subjects (Majander et al., 2016). For peripapillary RNFL measurement a 3.5-mm-diameter circular scan centered on the optic disc was used and the data for six sectors were collected.

### 2.3 Electrophysiology investigations

Twelve subjects underwent electrophysiological testing including pattern reversal and flash visual evoked potential (PVEP; FVEP) and pattern electroretinography (PERG), incorporating the standards of the International Society for Clinical Electrophysiology of Vision (ISCEV; Odom et al., 2010, Bach et al., 2013). Pattern ERGs were recorded to a 0.8- degree check size using both a standard checkerboard field (12 × 15°) and additionally to a large field (24 × 30°; LF PERG; Lenassi et al., 2012). The full-field photopic negative response (PhNR) was additionally recorded in 7 cases using diffuse red flash stimulation (640 nm) at 5 flash strengths (0.5, 1.0, 2.0, 5.0 and 10.0 cd·s·m^− 2^), superimposed on a blue background (450 nm; 2.25 cd·m^− 2^). Gold foil electrodes were used and the results compared to normative data.

### 2.4 Psychophysical investigations

#### 2.4.1 L- and S-cone critical flicker fusion and L-cone temporal contrast sensitivity

L- and S-cone temporal acuities (critical flicker fusion, CFF) and L-cone temporal contrast sensitivity functions (TCSFs) were measured using a Maxwellian-view optical system described in more detail elsewhere (Stockman et al., 2014a, Stockman et al., 2005). Predominantly L-cone or S-cone stimuli were used for the CFF measurements. The L-cone stimulus was produced by flickering a 650-nm circular target of 4° visual angle in diameter superimposed in the center of a steady 481-nm circular background field of 9° diameter. The background radiance was fixed at 8.3 log_10_ quanta s^− 1^ deg^− 2^ and the target radiance was varied in steps from 6.5 to 10.5 log_10_ quanta s^− 1^ deg^− 2^. The S-cone stimulus was produced by flickering a 440-nm circular target also of 4° diameter superimposed in the center of a 620-nm circular background field of 9° diameter. The 620-nm background radiance was fixed at 8.3 log_10_ quanta s^− 1^ deg^− 2^ and the 440-nm target radiance was varied in steps from 6.5 to 10 log_10_ quanta s^− 1^ deg^− 2^. The 650-nm target and 481-nm background were also used for the L-cone TCSF measurements with the time-averaged mean radiance of the 650-nm target fixed at 10.6 log_10_ quanta s^− 1^deg^− 2^. Before each run the subjects were light-adapted to the background and target for at least 2 min. The observers viewed the stimuli monocularly with their preferred eye (see Table 1) and interacted with the computer by means of an 8-button keypad. Each experiment was repeated 2–3 times on the same day. The method of adjustment was used. In the CFF measurements, observers varied the frequency of the 650 or 440-nm target, which was sinusoidally-flickering with a contrast of 92%, to find the frequency at each target radiance at which the flicker until the flicker just disappeared. In the TCSF measurements, observers varied the contrast of the sinusoidally-flickering 650-nm target to establish the contrast at each target frequency at which the flicker disappeared. Details of these measurements have been given elsewhere (Stockman et al., 2014a).

#### 2.4.2 Achromatic spatial contrast sensitivity function (SCSF)

Achromatic spatial contrast sensitivity (SCSF) was measured as a function of spatial frequency. The target stimuli were generated on a gamma-corrected Sony Trinitron monitor (Model GDM F520) with a resolution of 1600 × 1200 pixels and produced at a frame rate of 85-Hz. The monitor was driven by a DataPixx video processor (VPixx Technologies Inc., Saint-Bruno, QC, Canada). The full screen subtended a visual angle of 39° × 29° at a test distance of 0.57 m. The experiments were performed at a constant mean luminance of 44.57 cd/m^2^ as measured by a ColorCal calibration device (Cambridge Research Systems Ltd., Rochester, UK). The stimuli were horizontally-orientated Gabor patterns with spatial frequencies ranging from 0.25 to 16 cycles per degree (cpd) and with a spatial Gaussian window with a standard deviation of 6°. The target was presented for 500 ms, preceded and followed by 100 ms cosine-windowed onsets and offsets. The order of presentation was from low to high spatial frequency. Thresholds were measured using a staircase procedure. Observers indicated whether or not they could detect the spatial variation in the Gabor pattern using a 2-button keypad.

Stimulus contrast was defined as (L_max_ − L_min_) / (L_max_ + L_min_), where L_max_ and L_min_ are the maximum and minimum luminances in the Gabor pattern, respectively. Contrast was adjusted according to a one-up-one-down staircase procedure with a variable step-size. The step-size was 0.3 log_10_ units for the first five reversals (changes in the direction of the staircase), after which it was reduced to 0.2 log_10_ units for two more reversals and then finally to 0.1 log_10_ unit for the last four reversals. A single run required a total of 9 reversals, with the contrast “threshold” taken as the average of the last six reversals. Contrast sensitivity is the reciprocal of the contrast threshold. The SCSF measurements lasted approximately 20 min for each observer. The stimuli were viewed binocularly with natural pupils and appropriate correction if needed.

#### 2.4.3 Chromatic discrimination

Chromatic discrimination was tested using the so-called trivector test procedure implemented as part of the Cambridge Color Test (CCT), v1.5, (Cambridge Research Systems Ltd., Rochester, UK). The test was performed using a second gamma-corrected Sony FD Trinitron color monitor (Model GDM-F500R) connected to a VSG 2/5 visual stimulus generator (Cambridge Research Systems, Rochester, UK) with 800 by 600 pixel resolution. The CRT phosphors measured in CIE 1976 u′, v′ chromaticity coordinates (or CIE 1931 x, y coordinates) using ColorCal photometer (Cambridge Research System) were: red phosphor (R) u′ = 0.416; v′ = 0.522 (x = 0.610, y = 0.340); green phosphor (G) u′ = 0.117; v′ = 0.559 (x = 0.280, y = 0.595); blue phosphor (B) u′ = 0.159; v′ = 0.177 (x = 0.142, y = 0.070). The visual stimuli consisted of Landolt “C” targets that varied in orientation presented on a background of neutral chromaticity (CIE 1976 coordinates u′ = 0.1977, v′ = 0.4689). The background and the target were made up of small disks of variable size and luminance (ten equal steps between 8.0 and 18.0 cd·m^2^). The circles making up the Landolt C were varied in chromaticity to find the threshold for correctly discriminating the orientation of the Landolt C. The chromaticity was varied separately along the three color directions that are invisible to observers lacking L-, M- or S-cones; that is, the protan, deutan and tritan vector directions, respectively.

The test conditions were modified for observers with reduced visual acuity. Instead of the viewing distance being set so that the Landolt “C” opening subtended 1° of visual angle, the viewing distance was reduced to 62.6 cm so that the opening subtended 5° of visual angle. Two (A7, A9) of the 11 affected LHON carriers, and one patient A6 after spontaneous recovery, were able to perform the CCT test at this viewing distance. Another 5 patients were able to perform the test at a viewing distance of 5 to 10 cm that enabled recognition of the orientation of the gap despite their central scotomatas. Thresholds were measured along the protan, deutan, and tritan confusion lines. The upper limit for target saturation was increased to 1600 × 10^− 4^ u′v′ units and the number of steps increased from 6 to 10 in order to maintain the standard unit difference between the steps. The time allowed for each subject to respond was increased from the standard 8 s to 20 s. The test was then run using a standard descending psychophysical staircase procedure with six reversals. Observers were instructed to respond to the gap position (four-alternative forced choice) by means of a 4-button keypad. The test was performed binocularly with appropriate near corrections if needed. Six of the affected LHON carriers repeated the CCT test 6 to 12 months after their first visit.

### 2.5 Statistical analyses

Mann-Whitney *U* independent samples test was used for comparison of distribution of continuous variables in LHON and normal observers. Spearman's rank correlation test was used for the analysis of statistical dependence between the variables.

## 3 Results

### 3.1 The acute phase of LHON is characterized by rapid loss of RGCs

**Fig. 1:**
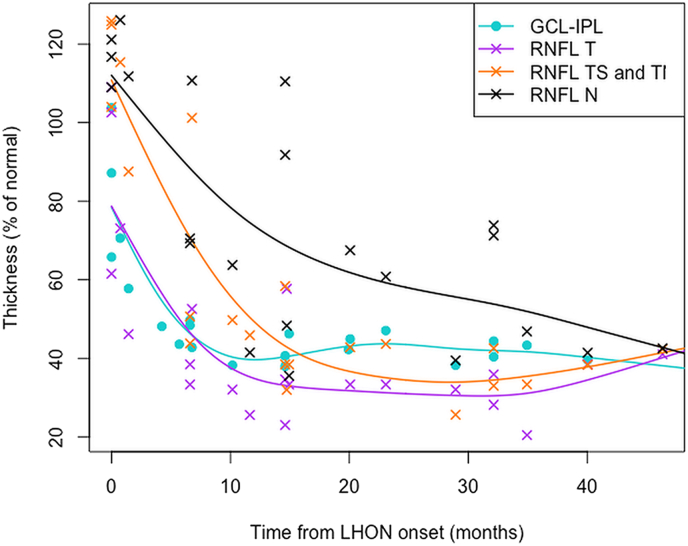
Optical coherence tomography data. Macular ganglion cell – inner plexiform layer complex (GCL-IPL) and peripapillary retinal nerve fiber layer (RNFL) thicknesses of 22 eyes have been presented as percentages of the normal mean and plotted as a function of time from LHON onset in each eye. The data have been shown for temporal (T), nasal (N) and combined supero- and inferotemporal (TS and TI) sectors of peripapillary RNFL. Generalized additive model (GAM) fits to data are indicated by the color coded lines. (For interpretation of the references to color in this figure legend, the reader is referred to the web version of this article.)

Macular SD-OCT revealed selective loss of the GCL-IPL complex thickness (50% of the normal) within the first few months of disease onset in LHON as shown in the Supplementary Table S1 and Fig. 1. This occurred in parallel with loss of the temporal peripapillary RNFL thickness (Spearman *rho* = 0.628, p = 0.002), whereas progression of RNFL thinning in the nasal sectors was less steep. The INL was slightly, but significantly thicker in the affected LHON carriers compared with controls or the unaffected LHON carriers.

### 3.2 Visual electrophysiology: pattern responses were more severely affected than photopic negative ERG responses

**Fig. 2:**
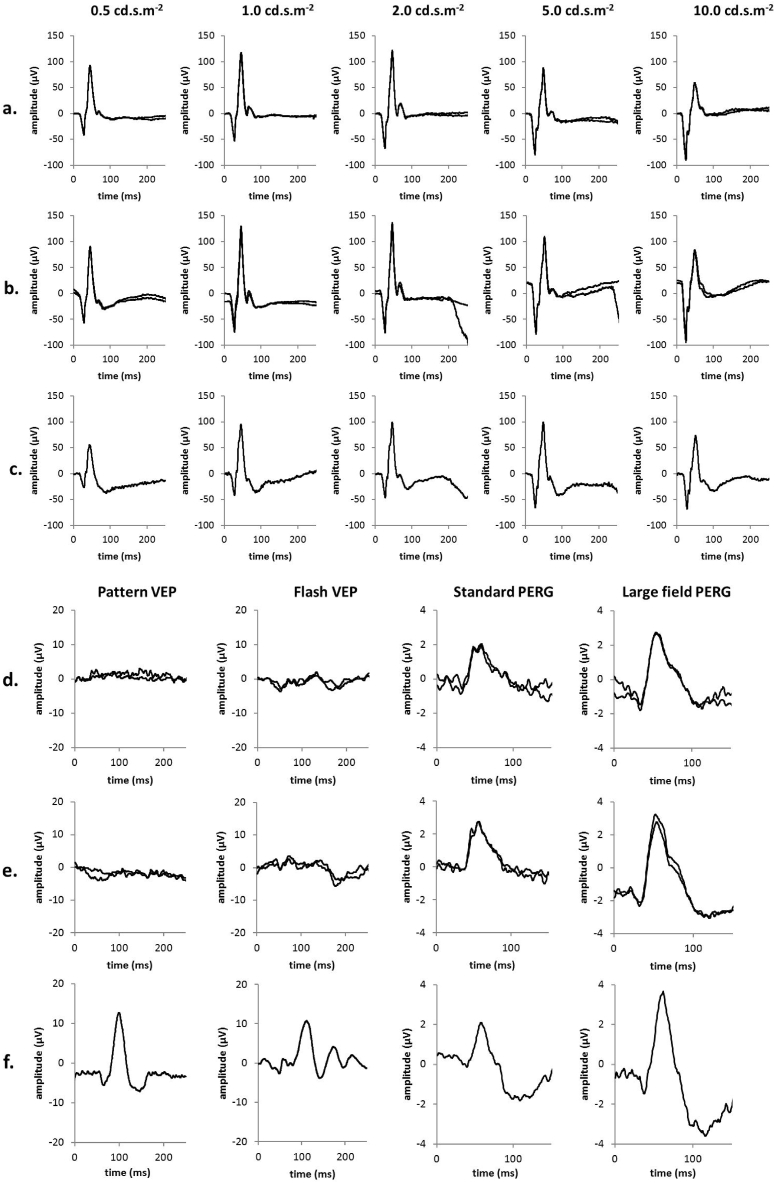
Photopic negative responses from the right (a) and left (b) eyes of a patient with LHON (A3). For comparison, representative normal recordings over a range of flash strengths (0.5–10.0 unit) have been provided (c). Pattern reversal VEPs, flash VEPs and pattern ERGs are shown for the right (d) and left (e) eyes of Patient A3 compared with representative normal recordings (f). The patient's recordings have been superimposed for all the parameters tested to demonstrate reproducibility.

The age of the patients at the time of electrophysiological testing ranged from 7.5 to 59.0 years (mean = 26.6 years; SD = 16.7 years). The duration of symptoms ranged from 0 to 27 months (mean 12.5 months). Visual acuity was severely impaired in all cases (range = 1.3 logMAR to perception of light). Characteristic electrophysiology waveforms for affected LHON carriers are shown in Fig. 2. Photopic negative responses were attenuated at all stimulus strengths (see corresponding plots in Fig. 2c and d; orange circles). Pattern reversal VEPs were undetectable and flash VEPs were grossly abnormal. Pattern ERG P50 is of short peak time and the waveforms had a low N95:P50 ratio, in keeping with severe optic nerve/retinal ganglion cell dysfunction bilaterally.

Pattern reversal VEPs were undetectable in 20 of 24 eyes and were delayed in 4 eyes of 4 subjects with detectable responses (peak time 121-142 ms; upper limit of normal 115 ms); amplitudes in 2 of the eyes with a delayed VEP were within the normal range (> 4 μV). Flash VEPs were undetectable in 5 eyes of 3 subjects (all with undetectable pattern VEP), were delayed (P2 > 140 ms) in 5 eyes, and were present but subnormal (< 5 μV) in 20 eyes. Flash VEPs were normal in 3 eyes of 2 subjects including one of the youngest individuals (age 7.5 years). The severity of pattern and flash VEP abnormality did not significantly correlate either with age or duration of symptoms.

**Fig. 3:**
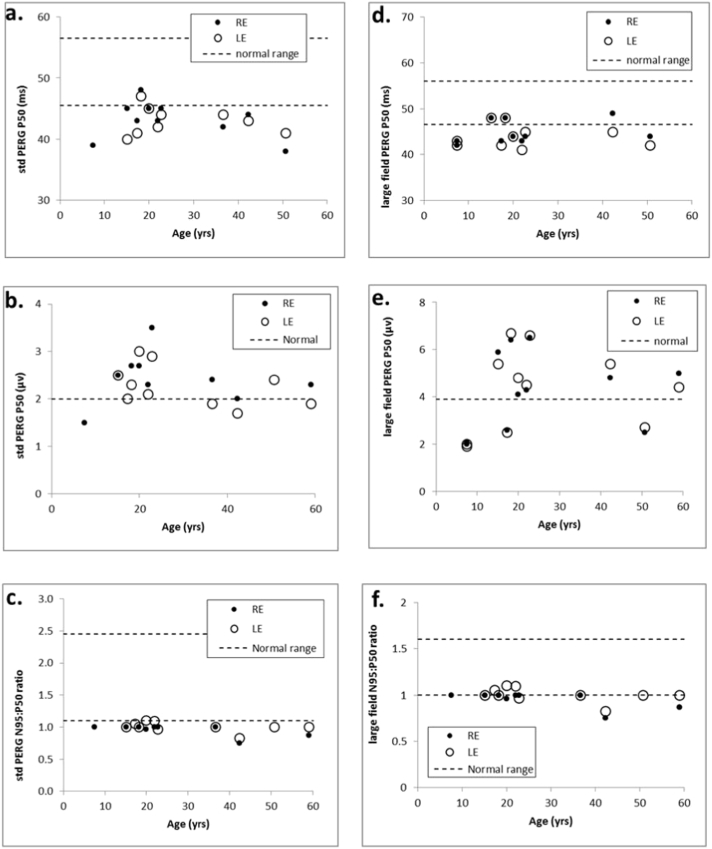
Pattern ERG data. Pattern ERG parameters were obtained to a standard (12° × 15°; a–c) and large (24 × 30°; d–f) checkerboard stimulus field for right and left eyes (RE; LE). Broken lines show the limits of normality defined as the minimum/maximum amplitude or peak time in a healthy cohort of subjects +/− the reference interval (maximum normal value-minimum normal value). Pattern ERG peak times and/or the N95:P50 ratio were abnormal in all the cases. Large field pattern ERG parameters were abnormal in most cases, including those in which PERG to the smaller field was excluded. The P50 amplitude of the pattern ERG was normal or near-normal in most cases, indicating good fixation despite of poor visual acuity.

Pattern ERG P50 to a standard stimulus (field size 12° × 15°) was of abnormally short peak time (< 45.5 ms) in 19 of 21 eyes (Fig. 3a PERG). P50 was of normal amplitude in most, mildly subnormal (< 2.0 μV) in 4 eyes of 4 patients (1.5–1.9 μV) and of borderline amplitude (2.0 μV) in a further 2 eyes (Fig. 3b PERG); P50 was of shortened peak time in all 6 eyes with a borderline or mildly subnormal amplitude. The N95:P50 amplitude ratio was subnormal (< 1.1) in 17 of 19 eyes (0.75–1.0) and at the lower limit of normal (ratio = 1.1) in a further 2 eyes (mean and median ratio of all cases 1.0; SD 0.1; Fig. 3c PERG). PERGs were technically poor in 5 eyes of 4 cases due to variable fixation (N = 1); pupil dilation (N = 1) or physiological noise and those eyes were excluded from analysis. PERG to a doubled stimulus field (24° × 30°) were obtained in 11 patients including 4 eyes in which the standard field PERG was excluded. Data were compared with a normative data set obtained using the same large field stimulus. Sixteen of 22 eyes were of abnormally short peak time (< 46.5 ms; Fig. 3d PERG) and these included 4 patients with additional bilateral P50 reduction (1.9 μV–2.7 μV; normal > 3.9 μV). The N95:P50 ratio was subnormal in 8, borderline in 10 and normal in 5 eyes (Fig. 3e PERG). There was no significant correlation between pattern ERG parameters and age or duration of symptoms.

**Fig. 4:**
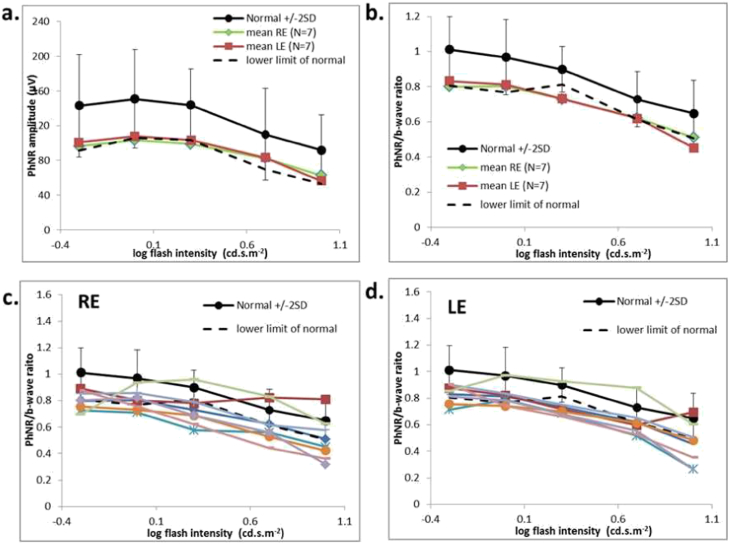
Photopic negative response (PhNR) data. Mean photopic negative responses (a) and the mean PhNR/b-wave ratio (b) were recorded in 7 individuals with LHON and compared with normal values at 5 flash strengths. The solid line and error bars show the mean normal values and 2 standard deviations from the mean. Broken lines show the limits of normality defined as the minimum amplitude in the normal cohort minus the 5% of the reference interval (maximum normal value-minimum normal value). Ratios from each individual are shown for right (RE; c) and left (LE; d) eyes. Sixty percent of responses were just outside the limits of normality (see text for details).

International-standard full-field ERGs revealed no clinically significant abnormality in the 7 individuals tested. PhNRs were recorded from 14 eyes of 7 cases, including 5 individuals that did not undergo standard full-field ERGs. The ratios of PhNR/b-wave ratios were within the normal range in 1 eye at all 5 flash intensities; in others the ratio was subnormal to one flash strength (N = 2 eyes), two (N = 3 eyes), three (N = 1 eye), four (N = 4 eyes) or 5 flash strengths (N = 3 eyes) (Fig. 4 PhNR). The mean magnitude of reduction of subnormal responses was 13% (range 1.5–23%) compared with the lower limit of normal. Forty percent of responses showed no abnormality (Fig. 4c and d).

### 3.3 Visual psychophysics

#### 3.3.1 L-cone temporal functions were severely compromised in LHON

**Fig. 5:**
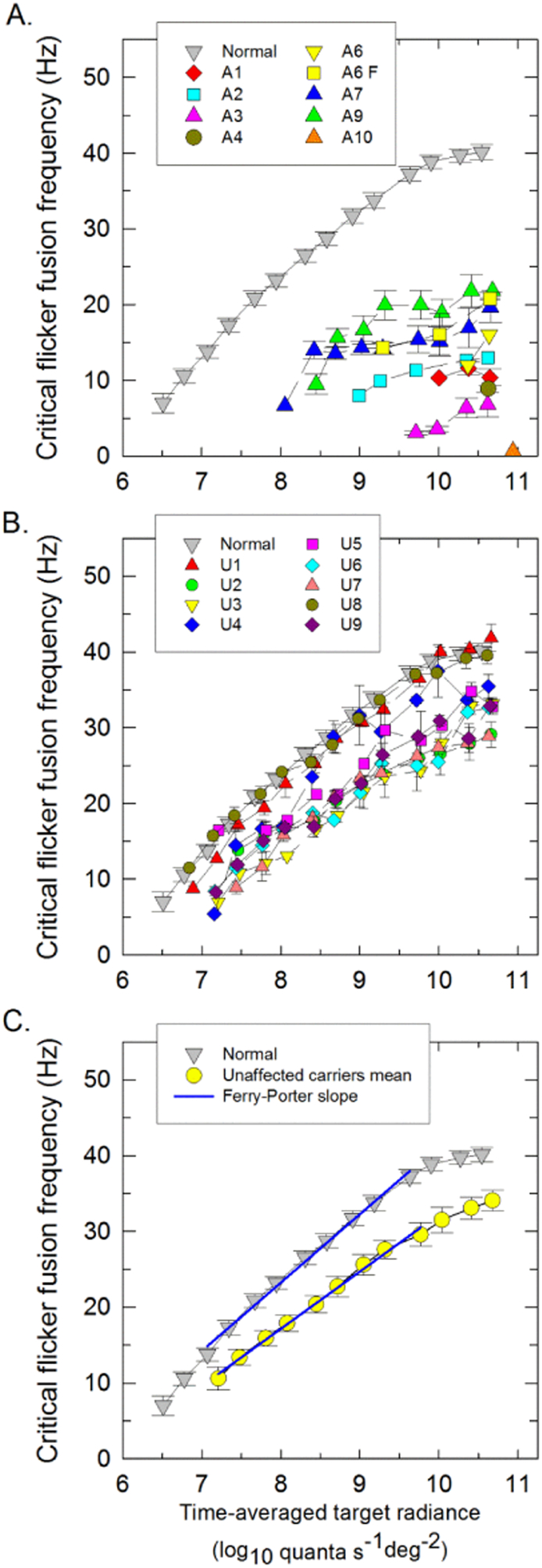
L-cone critical flicker fusion. L-cone critical flicker frequencies (cff) were measured on a 481–nm background of 8.26 log_10_ quanta s^− 1^ deg^− 2^ and plotted as a function of the mean log_10_ radiance of a 650-nm flickering target for the affected (A) and unaffected (B) LHON carriers. The mean cff data for 15 normal observers are represented by the grey triangles in all panels. The yellow squares (A6 F) indicate follow-up data for Patient A6, which were obtained after spontaneous recovery of best corrected visual acuity from 1.6 logMAR to 0.1 logMAR in the right eye. (C) Mean L-cone cff data for all unaffected LHON carriers (yellow circles)were compared with the mean normal data (grey triangles). The best-fitting Ferry-Porter slopes are indicated by the blue lines. The error bars represent ± 1 SEM either between runs for the individual patients or between observers for the mean data. (For interpretation of the references to color in this figure legend, the reader is referred to the web version of this article.)

The data for L-cone cff (temporal acuity) are presented in Fig. 5 and are summarized in the Supplementary Table S2. In the normal observers (grey triangles), flicker was first seen at a target radiance of 6.6 log_10_ quanta s^− 1^ deg^− 2^. The cff then grew linearly with log radiance over about 3 log_10_ units with a slope of 9.2 Hz per log_10_ unit (see upper blue line in the lower panel) until reaching a plateau near 40 Hz (Stockman et al., 2014a). The linear relationship between cff and log radiance is known as the Ferry-Porter law (Ferry, 1892, Porter, 1902). Eight of the 11 affected LHON carriers were able to detect L-cone flicker, but the mean radiance of 9.6 log_10_ quanta s^− 1^ deg^− 2^ at which flicker was first seen was 30 times higher than that for normal observers (Table S2). Three patients could detect flicker only at the highest target radiances. LHON patients thus required higher radiances to detect flicker than normal subjects and their cffs showed severe losses reaching a mean plateau of only 12 Hz, 28 Hz less than normal subjects. The lowest radiance at which unaffected LHON carriers first detected flicker was slightly higher than that for normal subjects (Table S2). Three carriers (U2, U6, U9) had significantly shallower Ferry-Porter slopes than normal and only three carriers (U1, U4, U8) had cffs that reached the normal plateau level of 40 Hz. The majority of the LHON carriers showed some loss of cff. Patient A6 experienced spontaneous recovery of BCVA from 1.6 logMAR to 0.1 logMAR between 6 and 18 months after LHON onset. His cff improved, but only marginally (A6 F, yellow squares).

**Fig. 6:**
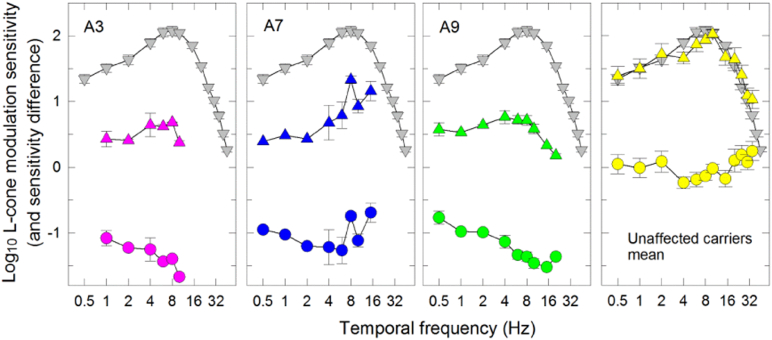
Log_10_ L-cone temporal contrast sensitivity. Log_10_ L-cone TCSFs were measured using a sinusoidally-modulated 650-nm target with a time-averaged mean radiance of 10.28 log_10_ quanta s^− 1^ deg^− 2^ superimposed on a 481-nm background of 8.29 log_10_ quanta s^− 1^ deg^− 2^ plotted as a function temporal frequency (logarithmic axis) for three affected LHON carriers (A3, A7, A9) (pink, blue and green triangles, respectively) and for the mean of 9 unaffected LHON carriers (yellow triangles). The mean TCSFs for 12 normal observers have been shown as grey triangles. The error bars represent ± 1 SEM either between runs for the individual patients or between subjects for the mean data. The mean difference in log sensitivity between each affected or the mean of unaffected LHON carrier and normal subjects are shown in the lower part of each panel (circles). (For interpretation of the references to color in this figure legend, the reader is referred to the web version of this article.)

Fig. 6 shows the L-cone temporal contrast sensitivity data. The mean L-cone TCSF for normal subjects peaks in sensitivity near 8 Hz and falls off in sensitivity at both low and high temporal frequencies (Stockman et al., 2014a, Stockman et al., 2014b, Ripamonti et al., 2014). Only patients A3, A7 and A9 of the affected carriers were able to perform the test and showed a mean sensitivity loss of 1.0 log_10_ unit. Unaffected LHON carriers had normal L-cone temporal modulation sensitivities but were unable to make settings at the highest temporal frequencies, consistent with their lower cffs.

#### 3.3.2 S-cone temporal function was unmeasurable in most LHON patients and reduced in the unaffected m.3460G > A carriers

**Fig. 7:**
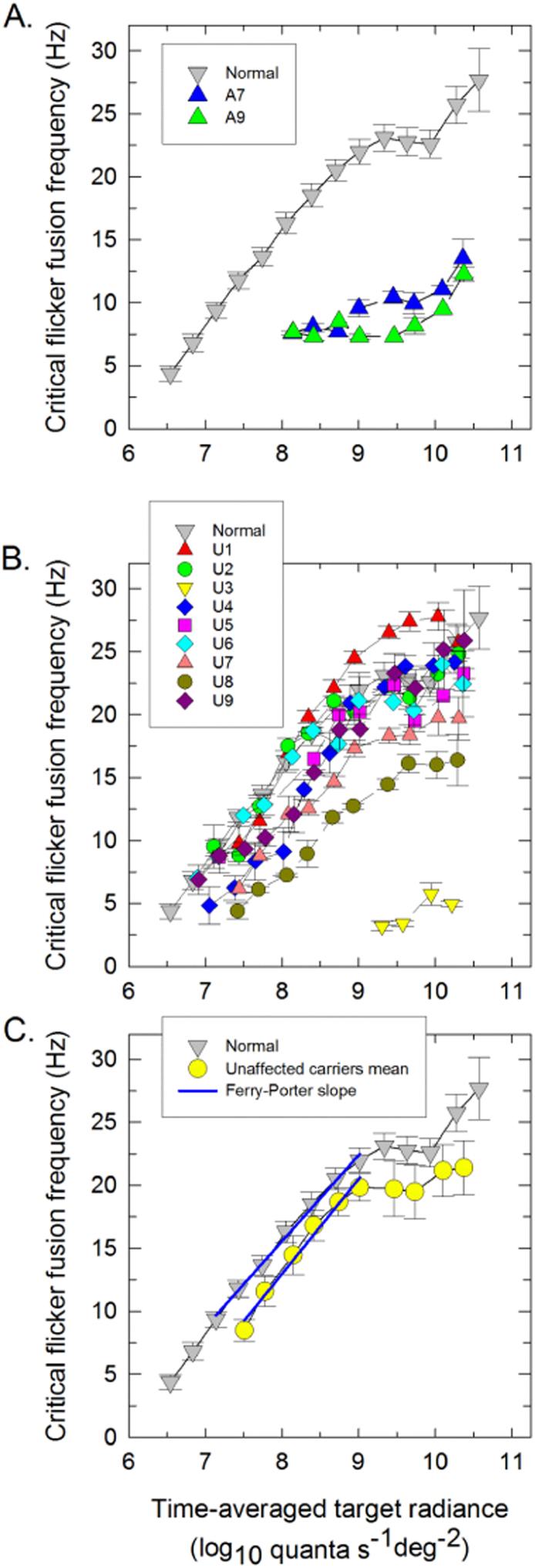
S-cone critical flicker frequencies (cff). This parameter was measured on a 9° 620-nm background of 11.41 log_10_ quanta s^−^^1^°deg^− 2^ and plotted as a function of the time-averaged mean log_10_ radiance of a 440-nm flickering target for each affected (A) and unaffected (B) LHON carrier (colored symbols). The mean cff data for 15 normal subjects are shown by the grey triangles in all panels. (C). Mean S-cone cff data for all unaffected LHON carriers (yellow circles) compared with the mean normal data (grey triangle). The best-fitting Ferry-Porter slopes are indicated by the blue lines (C). The error bars represent ± 1 SEM either between runs for the individual patients, or between subjects for the mean data. (For interpretation of the references to color in this figure legend, the reader is referred to the web version of this article.)

In the normal subjects, flicker was first seen at a target radiance of about 6.5 log_10_ quanta s^− 1^ deg^− 2^. Thereafter, the cff grows linearly with log radiance with a slope of 7.3 Hz per log_10_ unit, consistent with the Ferry-Porter law, until reaching a plateau at 9.0 log_10_ quanta s^− 1^ deg^− 2^ (see Fig. 7 and the Supplementary Table S3). The rise after 9.9 log_10_ quanta s^− 1^ deg^− 2^ is due to M-cone intrusion - the M-cones become more sensitive than S-cones at high radiances and thus mediate flicker detection (Stockman and Plummer, 1998, Stockman et al., 2014a).

Only two affected LHON carriers (A7, A9) were able to detect any 440-nm flicker, and all showed severely impaired temporal acuity. S-cone cff was relatively normal for the unaffected carriers of the m. 11778G > A and the m. 14484T > C mutations, who first saw flicker at 07.10 ± 0.06 (mean ± 1 SEM) log_10_ quanta s^− 1^ deg^− 2^, and exhibited a Ferry-Porter slope of 7.97 ± 0.39 Hz per decade (mean ± 1 SEM) and a cff plateau frequency of 22.94 ± 0.57 Hz (mean ± 1 SEM). In contrast, S-cone cff for the three carriers harboring the m.3460G > A mutation was compromised: those subjects first saw flicker at a radiance of 8.06 ± 0.51 log_10_ quanta s^− 1^ deg^− 2^, and exhibited a shallow Ferry-Porter slope of 5.03 ± 1.05 Hz per log_10_ unit, the cff reached a plateau frequency of 12.61 ± 3.80 Hz. One carrier of the three with the m.3460G > A mutation could detect flicker only at the highest radiances, but was within normal limits for the tritan measurements in the CCT (see below).

#### 3.3.3 Achromatic spatial contrast sensitivity function (SCSF) was unmeasurable in most LHON patients and mildly subnormal in the unaffected carriers

**Fig S1:**
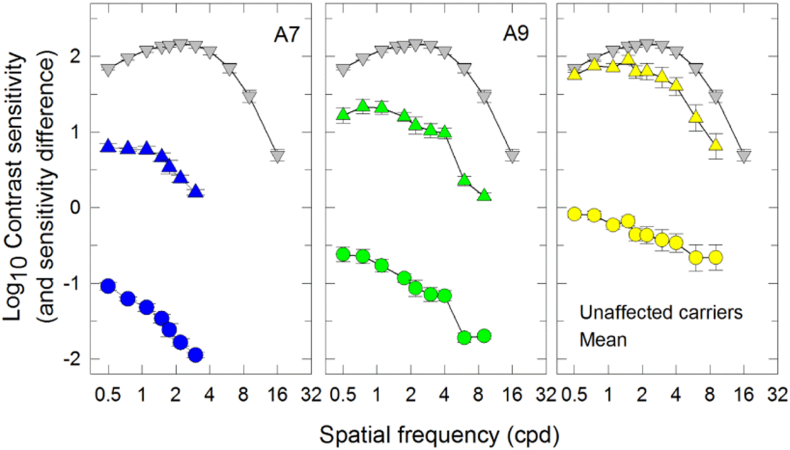
Achromatic spatial contrast sensitivity functions. CSFs expressed as log_10_ sensitivity as a function of spatial frequency (cycles per degree, cpd – logarithmic scale) are shown for two affected LHON carriers (A7, A9) (blue triangles), for the mean of the 9 unaffected LHON carriers (yellow triangles) and for normal controls (inverted grey triangles). The difference in sensitivity between LHON carriers and normal is also indicated in each panel by colored circles. The symbols and error bars represent the mean ± 1 SEM across normal observers, repeated runs of individual LHON patients or across 9 unaffected LHON carriers.

The mean achromatic spatial contrast sensitivity function for normal subjects is shown as inverted grey triangles in each of the three panels of Supplementary Fig. S1. The function is band-pass in shape peaking at 2 cpd and falling off in sensitivity at lower and higher spatial frequencies characteristic of other SCSFs measured at moderate and high intensities (Robson, 1966, van Nes and Bouman, 1967). Only two (A7 and A9) of the 11 affected LHON carriers could perform this test. Both patients showed a loss of contrast sensitivity that increased with frequency. In the unaffected LHON carriers, the achromatic SCSF was normal at the lowest spatial frequency but showed increasing loss as spatial frequency increased.

#### 3.3.4 Chromatic discrimination was severely affected in LHON

**Fig. 8:**
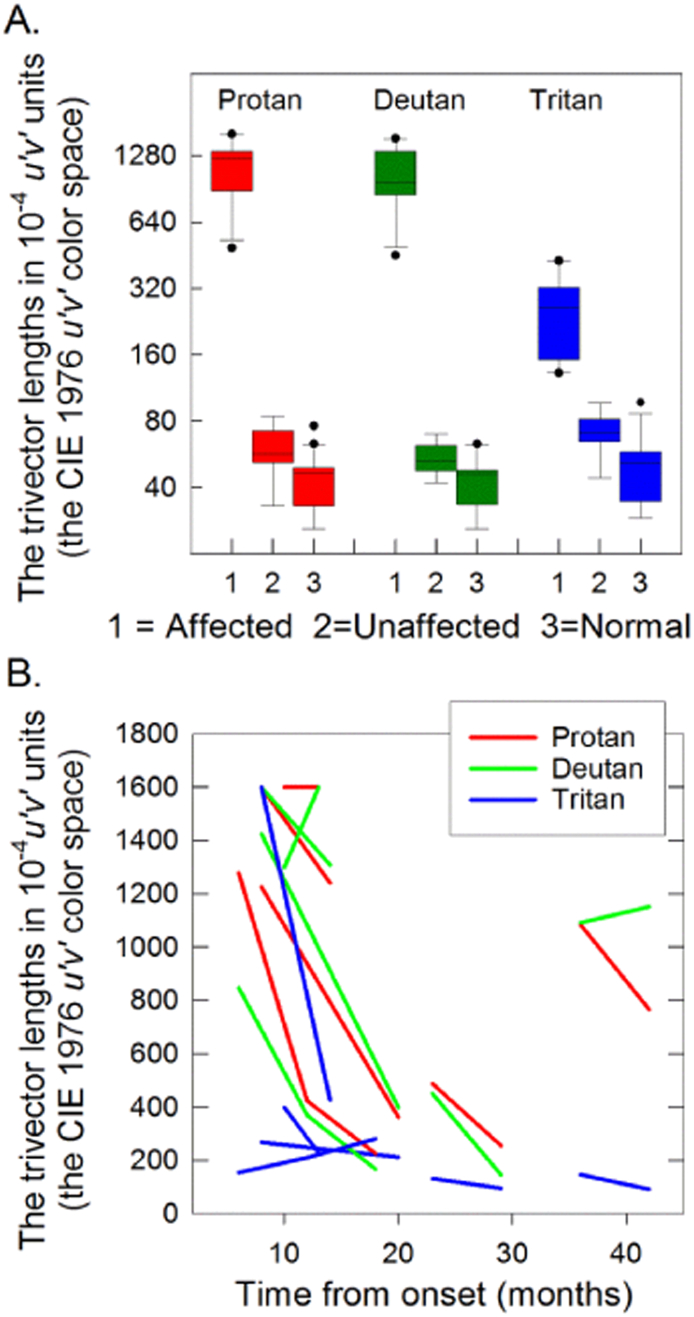
The Trivector Cambridge Color Test. (A) Box plots of the logarithmic vector lengths along the protan, deutan and tritan confusion lines expressed in 10^− 4^ u′v′ units (defined by the CIE 1976 u′v′ color space) for the affected (1, n = 8) and unaffected (2, n = 9) LHON carriers, and for normal observers (3, n = 15), showing median, range, inter-quartile range and outliers for all groups. (B) Changes in the vector lengths along the protan, deutan and tritan confusion lines have been expressed as 10^− 4^ u′v′ units for successive measurements of 6 affected LHON carriers and plotted as a function of time from disease onset.

Fig. 8A shows the vector lengths of the three confusion lines (protan, deutan, and tritan) of the Cambridge Color Test in 10^− 4^ u′v′ units (the CIE 1976 u′v′ color space). The longer the vector length the more saturated the color had to be for the orientation of the gap in the Landolt C to be discriminated. The maximum vector lengths were 1600 10^− 4^ u′v′ units (the CIE 1976 u′v′ color space). The vector lengths in the normal observers were comparable to those previously reported using standard targets with 1° gaps: 45.1 ± 1.0 for protan, 43.3 ± 0.8 for deutan and 51.5 ± 1.3 for tritan in 10^− 4^ u′v′ units (the CIE 1976 u′v′ color space) [mean ± SEM] (Paramei and Oakley, 2014). Altogether, 8 affected LHON carriers could discriminate the gap in the Landolt C, most viewed the CRT screen from only 5 to 10 cm Their vector thresholds along the protan and the deutan axes on their first tests were 25.8 ± 2.6 (mean ± SEM) and 25.4 ± 2.8 times higher, respectively, than controls whereas along the tritan axes the vectors were only 5.1 ± 0.7 times higher. Fig. 8B shows the vector lengths of the successive tests made on six affected LHON carriers. Three affected carriers (A1, A3, A6), initially studied 6 to 9 months after LHON onset, showed recovery of color discrimination, mainly along protan and deutan axes. Chromatic discrimination in unaffected LHON carriers was only marginally subnormal along all chromatic axes with high normal vector lengths. Affected observers therefore show a general loss along all three axes.

## 4 Discussion

Our comprehensive electrophysiological and psychophysical study of patients with acute and chronic LHON highlights the marked extent of RGC dysfunction in this classical mitochondrial optic neuropathy. Our study also indicates the utility of the standard transient pattern ERG technique in patients with LHON, with the recordings revealing severe central RGC dysfunction in all the patients tested, irrespective of visual acuity, age or the duration of symptoms. Detailed psychophysical data demonstrated loss of visual function in affected LHON carriers across a range of parameters, including achromatic spatial and L-cone temporal contrast sensitivity, L- and S-cone acuity, and chromatic discrimination. These findings are consistent with substantial losses of the principal RGC subtypes associated with each of the three major retinal pathways. Furthermore, with the exception of L-cone TCSF, unaffected LHON carriers also showed subclinical abnormalities in all the psychophysical measures that were evaluated.

A number of studies have reported abnormal cortical PR-VEPs responses in acute LHON, (Nikoskelainen et al., 1977, Hrynchak and Spafford, 1994, Mashima et al., 1997, Sharkawi et al., 2012, Ziccardi et al., 2013). Our PR-VEP results are consistent with these previous observations and the early involvement of the papillomacular bundle in this mitochondrial optic nerve disorder. The PERG reflects central retinal function and has two major components; the positive P50 and negative N95 (Holder, 1987). The N95 component arises in RGCs whereas approximately 30% of the P50 component originates in more anterior retinal structures (Holder, 1987, Holder, 2001, Holder, 2004, Ryan and Arden, 1988, Viswanathan et al., 2000). P50 is widely used to assess macular function whereas reduction in the N95:P50 ratio with preservation of P50 amplitude or shortening of P50 peak time suggests RGC dysfunction (Holder, 2001, Holder, 2004). However, reports of PERG findings in LHON have been contradictory (Sharkawi et al., 2012, Ziccardi et al., 2013, Jarc-Vidmar et al., 2015). Some studies of LHON have used surface electrodes and rapidly alternating stimuli to elicit steady-state PERG recordings (Lam et al., 2010, Lam et al., 2014) but the signal:noise ratio for surface recordings is lower than for corneal recordings and abnormal amplitude reduction does not distinguish macular from retinal ganglion cell dysfunction. Our study found consistent PERG abnormalities with shortened P50 peak time and reduced N95:P50 ratio in patients with acute LHON. Furthermore, P50 amplitude was preserved, consistent with adequate fixation despite the poor visual acuity and largely excluding a macular cause for the PR-VEP abnormality.

The photopic negative response (PhNR) component of the full-field electroretinogram is severely reduced in primates treated with tetrodotoxin and in experimental models of glaucoma, in keeping with a possible RGC origin (Viswanathan et al., 1999). The PhNR has also been shown to be attenuated in experimental and clinical studies of glaucoma and in other forms of optic neuropathies (Viswanathan et al., 1999, Gotoh et al., 2004, Sustar et al., 2009, North et al., 2010, Preiser et al., 2013, Machida, 2012, Morny et al., 2015). The majority of affected LHON patients in our study had responses near the limits of normal, with a substantial subgroup (40%) being within normal limits. There is no published data describing PhNRs in LHON, but mildly abnormal or borderline reductions in the PhNR have similarly been reported in dominant optic atrophy (Miyata et al., 2007). It is notable that both these mitochondrial optic neuropathies predominantly affect the papillomacular bundle with relative sparing of RGC axons in the retinal periphery (Johnston et al., 1979, Kerrison et al., 1995, Kjer et al., 1983, Sadun et al., 1994, Sadun et al., 2000). It should be noted that the full-field PhNR provides a measure of global RGC function and it could therefore be less sensitive in detecting focal RGC loss or dysfunction compared with the pattern ERG, which arises largely in central macular RGCs. Other methods such as focal PhNRs, involving flash stimulation of the central macular area, could potentially offer better sensitivity compared with full-field photopic negative responses, but with the disadvantage of generating comparatively smaller signals. These findings are pertinent to clinical trials of future therapeutic interventions, since an undetectable VEP cannot be used to monitor safety and PERG could prove the objective test of choice to monitor function of the most affected RGCs.

The international standard VEP and PERG stimuli involve high contrast checkerboard reversal stimuli that are suprathreshold, whereas psychophysics enables measurement of detection threshold. Affected LHON carriers in our study had impaired chromatic resolution in the protan and deutan axes. Their achromatic spatial sensitivity was also severely compromised. Only the two least affected patients (A7 and A9) were capable of performing the achromatic spatial CSF measurements, but even then, steep sensitivity losses with increasing spatial frequency were evident. These findings are consistent with the severe loss of the midget RGCs and the small calibre RGC axons found in post mortem histological studies (Sadun et al., 1994, Kerrison et al., 1995, Sadun et al., 2000). The majority of LHON patients in our study were able to detect a flickering long-wavelength light at high luminance levels, but with markedly reduced temporal resolution, in keeping with a severe deficit of magnocellular function. This previously unreported observation is pathologically relevant as it implies that the loss of parasol RGC function is also present in acute LHON, which is consistent with the LGN pathology reported in end-stage disease (Rizzo et al., 2012). Previous studies that have assessed the koniocellular pathway in LHON have been limited to color vision tests (Nikoskelainen et al., 1977). Only the least severely affected patients (A7 and A9) in our study were able to detect flickering short-wavelength light and additional tritan deficits in the CCT tests suggested severe koniocellular involvement. The smaller proportional loss found along the tritan axis compared with the protan and deutan axes in the CCT test of affected LHON carriers is of doubtful clinical significance, since the unusually close viewing distances adopted by 5 of the observers might have selectively reduced the tritan thresholds due to rod intrusion, retinal inhomogeneities or scatter. In addition, melanopsin-expressing RGCs, which are relatively preserved in LHON (Kawasaki et al., 2010, La Morgia et al., 2010), are preferentially sensitive to short-wavelength light (Lucas et al., 2014) and, in theory, may also contribute to blue color discrimination.

Previous studies on asymptomatic LHON carriers harboring the m.11778A > G mutation have reported subclinical visual impairment involving both the parvocellular and the magnocellular pathways as revealed by subtle chromatic and luminance contrast sensitivity deficits and impaired temporal processing (Ventura et al., 2005, Sadun et al., 2006, Ventura et al., 2007, Gualtieri et al., 2008, Mateus et al., 2015). Our study cohort included unaffected carriers with the m.3460A > G and m.14484T > C LHON mutations, in addition to m.11778A > G. Our data indicate that all three common mtDNA LHON mutations significantly impair achromatic spatial contrast sensitivity worst for higher spatial frequencies. The chromatic thresholds along all three confusion lines showed mild losses compared with normals highlighting dysfunction of the parvocellular pathway and confirming a previous report using similar methods (Mateus et al., 2015). The majority of LHON carriers also showed abnormalities of the long-wavelength temporal visual acuity, but with relatively minimal loss on the L-cone TCSF measurements. These parvocelluar and magnocellular related losses were present with all three common mtDNA LHON mutations. Unlike the long-wavelength sensitive temporal visual acuity, impairment of the short-wavelength temporal acuity was limited to the three unrelated unaffected carriers harboring the m.3460A > G mutation.

In conclusion, our study highlights the extent and severity of diffuse and focal electrophysiological measures of RGC dysfunction in LHON. PERG abnormalities in LHON are largely independent of age and can be elicited in patients with severely impaired visual acuity. Furthermore, psychophysical tests of achromatic and chromatic visual function suggest severe involvement or loss of midget, parasol and bistratified RGCs. These findings are highly relevant for the design of future clinical trials aimed at assessing therapeutic interventions and the viability of specific RGC subpopulations in patients affected with LHON.

The following are the supplementary data related to this article.

Table S1 Results of the macular and optic nerve head optical coherence tomography imaging. Table S1

Table S2 L-cone critical flicker fusion variables. Table S2

Table S3 S-cone critical flicker fusion variables. Table S3

## References

[bb0005] Akiyama H., Kashima T., Li D., Shimoda Y., Mukai R., Kishi S. (2013). Retinal ganglion cell analysis in Leber's hereditary optic neuropathy. Ophthalmology.

[bb0010] Bach M., Brigell M.G., Hawlina M., Holder G.E., Johnson M.A., McCulloch D.L., Meigen T., Viswanathan S. (2013). ISCEV standard for clinical pattern electroretinography (PERG): 2012 update. Doc. Ophthalmol..

[bb0015] Balducci N., Savini G., Cascavilla M.L., La Morgia C., Triolo G., Giglio R. (2015). Macular nerve fibre and ganglion cell layer changes in acute Leber's hereditary optic neuropathy. Br. J. Ophthalmol..

[bb0020] Barboni P., Savini G., Valentino M.L., Montagna P., Cortelli P., De Negri A.M. (2005). Retinal nerve fiber layer evaluation by optical coherence tomography in Leber's hereditary optic neuropathy. Ophthalmology.

[bb0025] Barboni P., Carbonelli M., Savini G., Ramos Cdo V., Carta A., Berezovsky A. (2010). Natural history of Leber's hereditary optic neuropathy: longitudinal analysis of the retinal nerve fiber layer by optical coherence tomography. Ophthalmology.

[bb0030] Carelli V., Rugolo M., Sgarbi G., Ghelli A., Zanna C., Baracca A. (2004). Bioenergetics shapes cellular death pathways in Leber's hereditary optic neuropathy: a model of mitochondrial neurodegeneration. Biochim. Biophys. Acta.

[bb0035] Carelli V., Ross-Cisneros F.N., Sadun A.A. (2004). Mitochondrial dysfunction as a cause of optic neuropathies. Prog. Retin. Eye Res..

[bb0040] Dacey D.M., Crook J.D., Packer O.S. (2014). Distinct synaptic mechanisms create parallel S-ON and S-OFF color opponent pathways in the primate retina. Vis. Neurosci..

[bb0050] Dowling J.E. (1987). The Retina, an Approachable Part of the Brain.

[bb0055] Ferry E.S. (1892). Persistence of vision. Am. J. Sci..

[bb0060] Gotoh Y., Machida S., Tazawa Y. (2004). Selective loss of the photopic negative response in patients with optic nerve atrophy. Arch. Ophthalmol..

[bb0065] Gualtieri M., Bandeira M., Hamer R.D., Costa M.F., Oliveira A.G.F., Moura A.L.A. (2008). Psychophysical analysis of contrast processing segregated into magnocellular and parvocellular systems in asymptomatic carriers of 11778 Leber's hereditary optic neuropathy. Vis. Neurosci..

[bb0070] Holder G.E. (1987). Significance of abnormal pattern electroretinography in anterior visual pathway dysfunction. Br. J. Ophthalmol..

[bb0075] Holder G.E. (2001). Pattern electroretinography (PERG) and an integrated approach to visual pathway diagnosis. Prog. Retin. Eye Res..

[bb0080] Holder G.E. (2004). Electrophysiological assessment of optic nerve disease. Eye.

[bb0085] Hrynchak P.K., Spafford M.M. (1994). Visual recovery in a patient with Leber hereditary optic neuropathy and the 14484 mutation. Optom. Vis. Sci..

[bb0090] Jarc-Vidmar M., Tajnik M., Brecelj J., Fakin A., Sustar M., Naji M. (2015). Clinical and electrophysiology findings in Slovene patients with Leber hereditary optic neuropathy. Doc. Ophthalmol..

[bb0095] Johnston P.B., Gaster R.N., Smith V.C., Tripathi R.C. (1979). A clinicopathological study of autosomal dominant optic atrophy. Am J. Ophthalmol..

[bb0100] Kawasaki A., Herbst K., Sander B., Milea D. (2010). Selective wavelength pupillometry in Leber hereditary optic neuropathy. Clin. Exp. Ophthalmol..

[bb0110] Kerrison J.B., Howell N., Miller N.R., Hirst L., Green W.R. (1995). Leber hereditary optic neuropathy. Electron microscopy and molecular genetic analysis of a case. Ophthalmology.

[bb0115] Kjer P., Jensen O.A., Klinken L. (1983). Histopathology of eye, optic nerve and brain in a case of dominant optic atrophy. Acta Opthalmol..

[bb0120] La Morgia C., Ross-Cisneros F.N., Sadun A.A., Hannibal J., Munarini A., Mantovani V. (2010). Melanopsin retinal ganglion cells are resistant to neurodegeneration in mitochondrial optic neuropathies. Brain.

[bb0125] Lam B.L., Feuer W.J., Abukhalil F., Porciatti V., Hauswirth W.W., Guy J. (2010). Leber hereditary optic neuropathy gene therapy clinical trial recruitment: year 1. Arch. Ophthalmol..

[bb0130] Lam B.L., Feuer W.J., Schiffman J.C., Porciatti V., Vandenbroucke R., Rosa P.R. (2014). Trial end points and natural history in patients with G11778A Leber hereditary optic neuropathy: preparation for gene therapy clinical trial. JAMA Ophthalmol..

[bb0135] Lee B.B., Martin P.R., Grünert U. (2010). Retinal connectivity and primate vision. Prog. Retin. Eye Res..

[bb0140] Lenassi E., Robson A.G., Hawlina M., Holder G.E. (2012). The value of two field PERG in routine clinical electrophysiological practice. Retina.

[bb0145] Lennie P., Movshon J.A. (2005). Coding of color and form in the geniculostriate visual pathway. J. Opt. Soc. Am. A Opt. Image Sci. Vis..

[bb0150] Levin L.A. (2015). Superoxide generation explains common features of optic neuropathies associated with cecocentral scotomas. J. Neuroophthalmol..

[bb0155] Lin C.S., Sharpley M.S., Fan W., Waymire K.G., Sadun A.A., Carelli V. (2012). Mouse mtDNA mutant model of Leber hereditary optic neuropathy. Proc. Natl. Acad. Sci. U. S. A..

[bb0160] Lucas R.J., Peirson S.N., Berson D.M., Brown T.M., Cooper H.M., Czeisler C.A. (2014). Measuring and using light in the melanopsin age. Trends Neurosci..

[bb0165] Machida S. (2012). Clinical applications of the photopic negative response to optic nerve and retinal diseases. J. Ophthalmol..

[bb0170] Mackey D.A., Oostra R.J., Rosenberg T., Nikoskelainen E., Bronte-Stewart J., Poulton J. (1996). Primary pathogenic mtDNA mutations in multigeneration pedigrees with Leber hereditary optic neuropathy. Am. J. Hum. Genet..

[bb0175] Majander A., Bitner-Glindzicz M., Chan C.M., Duncan H.J., Chinnery P.F., Subash M. (2016). Lamination of the outer plexiform layer in optic atrophy caused by dominant WFS1 mutations. Ophthalmology.

[bb0180] Mashima Y., Imamura Y., Oguchi Y. (1997). Dissociation of damage to spatial and luminance channels in early Leber's hereditary optic neuropathy manifested by the visual evoked potential. Eye (London)..

[bb0185] Mateus C., d'Almeida O.C., Reis A., Silva E., Castelo-Branco M. (2015). Genetically induced impairment of retinal ganglion cells at the axonal level is linked to extrastriate cortical plasticity. Brain Struct. Funct..

[bb0190] Miyata K., Nakamura M., Kondo M., Lin J., Ueno S., Miyake Y., Terasaki H. (2007). Reduction of oscillatory potentials and photopic negative response in patients with autosomal dominant optic atrophy with OPA1 mutations. Invest. Ophthalmol. Vis. Sci..

[bb0195] Mizoguchi A., Hashimoto Y., Shinmei Y., Nozaki M., Ishijima K., Tagawa Y., Ishida S. (2015). Macular thickness changes in a patient with Leber's hereditary optic neuropathy. BMC Ophthalmol..

[bb0200] Morny E.K., Margrain T.H., Binns A.M., Votruba M. (2015). Electrophysiological ON and OFF responses in autosomal dominant optic atrophy. Invest. Ophthalmol. Vis. Sci..

[bb0205] van Nes F.L., Bouman M.A. (1967). Spatial modulation transfer in the human eye. J. Opt. Soc. Am..

[bb0210] Nikoskelainen E., Sogg R.L., Rosenthal A.R., Friberg T.R., Dorfman L.J. (1977). The early phase in Leber hereditary optic atrophy. Arch. Ophthalmol..

[bb0215] Nikoskelainen E., Hoyt W.F., Nummelin K. (1982). Ophthalmoscopic findings in Leber's hereditary optic neuropathy. I. Fundus findings in asymptomatic family members. Arch. Ophthalmol..

[bb0220] Nikoskelainen E.K., Huoponen K., Juvonen V., Lamminen T., Nummelin K., Savontaus M.L. (1996). Ophthalmologic findings in Leber hereditary optic neuropathy, with special reference to mtDNA mutations. Ophthalmology.

[bb0225] North R.V., Jones A.L., Drasdo N., Wild J.M., Morgan J.E. (2010). Electrophysiological evidence of early functional damage in glaucoma and ocular hypertension. Invest. Ophthalmol. Vis. Sci..

[bb0230] Odom J.V., Bach M., Brigell M., Holder G.E., McCulloch D.L., Tormene A.P., Vaegan (2010). ISCEV standard for clinical visual evoked potentials (2009 update). Doc. Ophthalmol..

[bb0235] Paramei G.V., Oakley B. (2014). Variation of color discrimination across the life span. J. Opt. Soc. Am. A Opt. Image Sci. Vis..

[bb0240] Porter T.C. (1902). Contributions to the study of flicker. Paper II. Proc. R. Soc. Lond. Ser. A.

[bb0245] Preiser D., Lagrèze W.A., Bach M., Poloschek C.M. (2013). Photopic negative response versus pattern electroretinogram in early glaucoma. Invest. Ophthalmol. Vis. Sci..

[bb0250] Ripamonti C., Henning G.B., Ali R.R., Bainbridge J.W., Robbie S.J., Sundaram V. (2014). Nature of the visual loss in observers with Leber's congenital amaurosis caused by specific mutations in RPE65. Invest. Ophthalmol. Vis. Sci..

[bb9815] Rizzo G., Tozer K.R., Tonon C., Manners D., Testa C., Malucelli E. (2012). Secondary post-geniculate involvement in Leber's hereditary optic neuropathy. PLoS One.

[bb0255] Robson J.G. (1966). Spatial and temporal contrast sensitivity functions of the visual system. J. Opt. Soc. Am..

[bb0260] Rodieck R.W. (1998). The First Steps in Seeing.

[bb0265] Ryan S., Arden G.B. (1988). Electrophysiological discrimination between retinal and optic nerve disorders. Doc. Ophthalmol..

[bb0270] Sadun A.A., Kashima Y., Wurdeman A.E., Dao J., Heller K., Sherman J. (1994). Morphological findings in the visual system in a case of Leber's hereditary optic neuropathy. Clin. Neurosci..

[bb0275] Sadun A.A., Win P.H., Ross-Cisneros F.N., Walker S.O., Carelli V. (2000). Hereditary optic neuropathy differentially affects smaller axons in the optic nerve. Tr. Am. Ophth. Soc..

[bb0280] Sadun A.A., Salomao S.R., Berezovsky A., Sadun F., Denegri A.M., Quiros P.A. (2006). Subclinical carriers and conversions in Leber hereditary optic neuropathy: a prospective psychophysical study. Trans. Am. Ophthalmol. Soc..

[bb0285] Sadun A.A., Karanjia R., Pan B.X., Ross-Cisneros F.N., Carelli V. (2015). Reactive oxygen species in mitochondrial optic neuropathies: comment. J. Neuroophthalmol..

[bb0290] Sharkawi E., Oleszczuk J.D., Holder G.E., Raina J. (2012). Clinical and electrophysiological recovery in Leber hereditary optic neuropathy with G3460A mutation. Doc. Ophthalmol..

[bb0295] Stockman A., Plummer D.J. (1998). Color from invisible flicker: a failure of the Talbot-Plateau law caused by an early ‘hard’ saturating nonlinearity used to partition the human short-wave cone pathway. Vis. Res..

[bb0300] Stockman A., Plummer D.J., Montag E.D. (2005). Spectrally opponent inputs to the human luminance pathway: slow + M and − L cone inputs revealed by intense long-wavelength adaptation. J. Physiol..

[bb0305] Stockman A., Henning G.B., Michaelides M., Moore A.T., Webster A.R., Cammack J., Ripamonti C. (2014). Cone dystrophy with “supernormal” rod ERG: psychophysical testing shows comparable rod and cone temporal sensitivity losses with no gain in rod function. Invest. Ophthalmol. Vis. Sci..

[bb0310] Stockman A., Henning G.B., Moore A.T., Webster A.R., Michaelides M., Ripamonti C. (2014). Visual consequences of molecular changes in the guanylate cyclase-activating protein. Invest. Ophthalmol. Vis. Sci..

[bb0315] Sustar M., Cvenkel B., Brecelj J. (2009). The effect of broadband and monochromatic stimuli on the photopic negative response of the electroretinogram in normal subjects and in open-angle glaucoma patients. Doc. Ophthalmol..

[bb0320] Ventura D.F., Quiros P., Carelli V., Salomão S.R., Gualtieri M., Oliveira A.F.G. (2005). Chromatic and luminance contrast sensitivities in asymptomatic carriers from a large Brazilian pedigree of 11778 Leber Hereditary Optic Neuropathy. Invest. Ophthalmol. Vis. Sci..

[bb0325] Ventura D.F., Gualtieri M., Oliveira A.G., Costa M.F., Quiros P., Sadun F. (2007). Male prevalence of acquired color vision defects in asymptomatic carriers of Leber's hereditary optic neuropathy. Invest. Ophthalmol. Vis. Sci..

[bb0330] Viswanathan S., Frishman L.J., Robson J.G., Harwerth R.S., Smith E.L. (1999). The photopic negative response of the macaque electroretinogram: reduction by experimental glaucoma. Invest. Ophthalmol. Vis. Sci..

[bb0335] Viswanathan S., Frishman L.J., Robson J.G. (2000). The uniform field and pattern ERG in macaques with experimental glaucoma: removal of spiking activity. Invest. Ophthalmol. Vis. Sci..

[bb0345] Wässle H., Boycott B.B. (1991). Functional architecture of the mammalian retina. Physiol. Rev..

[bb0355] Yu-Wai-Man P., Chinnery P.F., Pagon R.A., Adam M.P., Ardinger H.H., Wallace S.E., Amemiya A., LJH Bean, Bird T.D., Fong C.T., Mefford H.C., RJH Smith, Stephens K. (2013). Leber hereditary optic neuropathy. GeneReviews® [Internet].

[bb0360] Yu-Wai-Man P., Votruba M., Moore A.T., Chinnery P.F. (2014). Treatment strategies for inherited optic neuropathies – past, present and future. Eye.

[bb0365] Zhang Y., Huang H., Wei S., Qiu H., Gong Y., Li H. (2014). Characterization of retinal nerve fiber layer thickness changes associated with Leber's hereditary optic neuropathy by optical coherence tomography. Exp. Ther. Med..

[bb0370] Ziccardi L., Sadun F., De Negri A.M., Barboni P., Savini G., Borrelli E. (2013). Retinal function and neural conduction along the visual pathways in affected and unaffected carriers with Leber's hereditary optic neuropathy. Invest. Ophthalmol. Vis. Sci..

[bb0375] Ziccardi L., Parisi V., Giannini D., Sadun F., De Negri A.M., Barboni P. (2015). Multifocal VEP provide electrophysiological evidence of predominant dysfunction of the optic nerve fibers derived from the central retina in Leber's hereditary optic neuropathy. Graefes Arch. Clin. Exp. Ophthalmol..

