# Supplementary material for: The pattern of retinal ganglion cell dysfunction in Leber hereditary optic neuropathy
Source: Mitochondrion. 2017 Sep;36:138–49. doi: 10.1016/j.mito.2017.07.006 (PMC5644721; doi:10.1016/j.mito.2017.07.006)
Supplement: Table S1 — Results of the macular and optic nerve head optical coherence tomography imaging. [file mmc1.docx]

**Table S1. Results of the macular and optic nerve head optical coherence tomography imaging.**

| Retinal layer | Layer thickness (µm) (mean±SD) | | | |
| --- | --- | --- | --- | --- |
|  | Affected LHON (n=26 eyes) | P * | Unaffected LHON (n=18 eyes) | Control (n=48 eyes) |
| Macula |  |  |  |  |
| Retina | 306.3 ± 24.8 | <0.001 | 349.0± 8.5 | 340.8 ± 13.3 |
| RNFL | 19.2 ± 2.8 | <0.001 | 24.8 ± 1.1 | 24.2 ± 2.1 |
| GCL-IPL | 47.1 ± 14.6 | <0.001 | 94.9 ± 3.4 | 93.5 ± 7.8 |
| INL | 45.1 ± 4.7 | <0.001 | 39.2 ± 2.3 | 39.6 ± 3.5 |
| OPL | 33.0 ± 8.2 | 0.955 | 34.9 ± 4.7 | 32.3 ± 4.0 |
| OPL-ONL | 110.9 ± 10.0 | <0.001 | 107.4 ± 8.1 | 102.0 ± 8.6 |
| Outer retina | 83.9 ± 3.4 | 0.002 | 82.9 ± 2.6 | 81.5 ± 2.7 |
| Peripapillary RNFL |  |  |  |  |
| Inferotemporal | 83.2 ± 47.3 |  | 152.4 ± 18.6 | 147** |
| Temporal | 33.4 ± 17.2 |  | 72.7 ± 2.5 | 78** |
| Superotemporal | 73.5 ± 46.0 |  | 122.3 ± 20.4 | 138** |
| Superonasal | 77.1 ± 35.2 |  | 111.4 ± 30.8 | 102** |
| Nasal | 54.0 ± 21.1 |  | 83.3 ± 13.7 | 72** |
| Inferonasal | 79.7 ± 35.0 |  | 126.4 ± 26.4 | 108** |
| Average | 66.8 ± 30.0 |  | 111.4 ± 12.4 | 108** |

Abbreviations: GCL-IPL, ganglion cell layer - inner plexiform layer complex; INL, inner nuclear layer; LHON, Leber hereditary optic neuropathy; OPL, outer plexiform layer; OPL-ONL, outer plexiform layer - outer nuclear layer complex; RNFL, retinal nerve fiber layer.

* Mann-Whitney *U* Test

** Heidelberg OCT normative mean
