# Supplementary material for: The pattern of retinal ganglion cell dysfunction in Leber hereditary optic neuropathy
Source: Mitochondrion. 2017 Sep;36:138–49. doi: 10.1016/j.mito.2017.07.006 (PMC5644721; doi:10.1016/j.mito.2017.07.006)
Supplement: Table S2 — L-cone critical flicker fusion variables. [file mmc2.docx]

**Table S2. L-cone critical flicker fusion variables.**

| **Observer** | **L-cone** | | | | |
| --- | --- | --- | --- | --- | --- |
|  | The lowest radiance  (log_10_ quanta s^-1^deg^-2^) | Ferry-Porter slope  Per decade R^2^ | | Frequency (Hz)  at 8.5 log_10_ quanta  s^-1^deg^-2^ radiance | Plateau frequency (Hz) at 10.3-10.6 log_10_ quanta  s^-1^deg^-2^ radiance |
| Normal mean±SE | 6.65±0.06 | 9.16±0.35 | 0.998 | 27.90±0.91 | 40.18±0.88 |
| LHON |  |  |  |  |  |
| A1 | 10.00 | 0.295 | 0.017 | ND | 11.60 |
| A2 | 8.99 | 2.873 | 0.933 | ND | 13.00 |
| A3 | 9.71 | 4.594 | 0.931 | ND | 6.78 |
| A4 | 10.63 | ND | ND | ND | 8.92 |
| A6 | 10.37 | ND | ND | ND | 16.00 |
| A7 | 8.05 | 2.914 | 0.762 | ND | 19.67 |
| A9 | 8.45 | 10.847 | 0.896 | ND | 21.84 |
| A10 | 10.94 | ND | ND | ND | 0.67 |
| A11 |  |  |  |  |  |
| LHON mean±SE | 9.64±0.43 | 4.30±1.59 | 0.71±0.18 |  | 12.31±2.84 |
| Mann-Whitney *U* test, p | <0.001 | <0.001 |  |  | <0.001 |
| Carrier |  |  |  |  |  |
| U1 | 6.89 | 8.62 | 0.99 | 26.08 | 41.89 |
| U2 | 7.46 | 5.37 | 0.97 | 19.32 | 29.11 |
| U3 | 7.21 | 7.85 | 0.99 | 17.02 | 33.17 |
| U4 | 5.42 | 9.24 | 0.92 | 24.13 | 37.22 |
| U5 | 7.21 | 8.20 | 0.94 | 21.34 | 34.80 |
| U6 | 7.16 | 6.55 | 0.93 | 18.83 | 30.72 |
| U7 | 7.43 | 9.30 | 0.99 | 19.07 | 28.89 |
| U8 | 6.84 | 8.00 | 0.99 | 27.21 | 39.56 |
| U9 | 7.18 | 7.17 | 0.95 | 19.48 | 32.89 |
| All carriers Mean±SE | 6.98±0.19 | 7.81±0.43 | 0.96±0.01 | 21.39±1.19 | 34.25±1.43 |
| Mann-Whitney *U* test, p | 0.004 | 0.035* |  | 0.001* | 0.004* |

Abbreviation: LHON, Leber hereditary optic neuropathy; ND, not determined.
