# Supplementary material for: The pattern of retinal ganglion cell dysfunction in Leber hereditary optic neuropathy
Source: Mitochondrion. 2017 Sep;36:138–49. doi: 10.1016/j.mito.2017.07.006 (PMC5644721; doi:10.1016/j.mito.2017.07.006)
Supplement: Table S3 — S-cone critical flicker fusion variables. [file mmc3.docx]

**Table S3. S-cone critical flicker fusion variables.**

| **Observer** | **S-cone** | | | | |
| --- | --- | --- | --- | --- | --- |
|  | The lowest radiance  (log_10_ quanta s^-1^deg^-2^) | Ferry-Porter slope  Per decade R^2^ | | Frequency (Hz)  at 8.5 log_10_ quanta  s^-1^deg^-2^ radiance | Plateau frequency (Hz) at 9.2-9.4 log_10_ quanta  s^-1^deg^-2^ radiance |
| Normal | 6.77±0.09 | 7.28±0.29 | 0.998 | 18.99±0.89 | 23.88±0.98 |
| Affected LHON |  |  |  |  |  |
| A7 | 8.13 | 2.20 | 0.8219 | ND | 10.44 |
| A9 | 8.14 | 0.1074 | 0.055 | ND | 7.63 |
| All affected | 8.14±0.00 | 4.30±1.59 |  |  | 9.04±0.53 |
| Unaffected |  |  |  |  |  |
| U1 | 7.44 | 10.12 | 0.99 | 20.50 | 26.50 |
| U2 | 7.11 | 7.27 | 0.91 | 18.80 | 22.25 |
| U3 | 9.31 | 2.57 | 0.70 | 1.06 | 3.39 |
| U4 | 7.05 | 9.66 | 0.95 | 16.07 | 22.17 |
| U5 | 7.18 | 6.14 | 0.98 | 17.15 | 22.33 |
| U6 | 6.89 | 6.30 | 0.94 | 17.93 | 21.11 |
| U7 | 7.44 | 6.97 | 0.98 | 14.62 | 18.33 |
| U8 | 7.42 | 5.64 | 0.98 | 10.33 | 16.11 |
| U9 TC | 6.91 | 6.39 | 0.91 | 15.86 | 23.28 |
| All unaffected | 7.42±0.23 | 6.78±0.74 | 0.95±0.01 | 14.70±1.95 | 19.50±2.11 |
| Mann-Whitney *U* test, p | 0.001* | 0.411 |  | 0.055 | 0.049 |

Abbreviation: LHON, Leber hereditary optic neuropathy; ND, not determined.
